# Recent Progress and Future Perspectives of Immunotherapy in Advanced Gastric Cancer

**DOI:** 10.3389/fimmu.2022.948647

**Published:** 2022-07-01

**Authors:** Xin Jin, Zhaorui Liu, Dongxiao Yang, Kai Yin, Xusheng Chang

**Affiliations:** ^1^ Department of Gastrointestinal Surgery, Changhai Hospital, Naval Medical University, Shanghai, China; ^2^ Key Laboratory of Natural Medicines of the Changbai Mountain, Ministry of Education, Yanbian University, Yanji, China

**Keywords:** advanced gastric cancer, immunotherapy, immune checkpoint inhibitor, adoptive cell therapy, cancer vaccine, CAR-T cell therapy

## Abstract

As one of the most common forms of solid tumours, gastric carcinoma has been revealed as the third leading cause of death worldwide. The symptom of gastric cancer is usually not obvious and thus difficult to detect at earlier stages. Therefore, gastric cancer is already in the advanced stage once detected in patients, which has a poor prognosis due to ineffective therapies and multiple resistance. Recent advance in understanding the microenvironment of cancer has significantly promoted the development of immunotherapy for advanced gastric cancer. Immunotherapy can induce immune responses in gastric cancer patients thus leads to the destruction of cancer cells. In comparison of traditional therapy, immunotherapy has demonstrated robust efficacy and tolerable toxicity. Therefore, this novel strategy for treatment of advanced gastric cancer has gain increasingly popularity. In this review, we summarize recent progress of immunotherapy in advanced gastric cancer, such as immune check point inhibitors, adoptive cell therapy, VEGF inhibitors, cancer vaccines and CAR-T cell therapy. We highlight immunotherapies involved in clinical applications and discuss the existing challenges of current immunotherapies and promising strategies to overcome these limitations.

## 1. Introduction

Gastric cancer is the third leading cause of cancer-related death (1). Due to the delay in diagnosis and lack of effective therapies, patients with advanced gastric cancer suffer from poor prognosis and a short lifespan of approximately one year (2). The commonly used therapies of advanced gastric cancer are radiotherapy, chemotherapy and targeted therapy. Agents such as imatinib, larotrectinib, entrectinib and regorafenib are widely used for treatment of advanced gastric cancer (3, 4). However, multi-drug resistance and tumour relapse have largely limited the effectiveness of these traditional therapies.

In the recent years, immunotherapy has become a novel therapy to treat advanced gastric cancer and has quickly drawn the attention of researchers around the world owing to its amazing anti-tumour efficacy (5, 6). A better understanding of the tumour microenvironment has greatly facilitated the development of immunotherapies in advance gastric cancer (7). The most widely applied immunotherapies against advanced gastric cancer including immune checkpoint inhibitors (ICIs), adoptive cell therapy, cancer vaccine, vascular endothelial growth factor A (VEGFA) antibody and chimeric antigen receptor (CAR) T therapy, etc (8–10). Studies have shown that ICIs such as anti-PD-1/PD-L1 antibodies could effectively kill cancer cells *via* activation of the immune response (11). Clinical trials of ICIs have displayed efficacy and safety for cancer patients (12, 13). Notably, several ICIs such as pembrolizumab, avelumab, sintilimab, tislelizumab and ipilimumab have been approved for clinical application in combination with targeted therapy for treatment of advanced gastric tumour (14, 15).

In this review, we describe state-of-the-art development of immunotherapy for treatment of advanced gastric cancer, highlighting recent advances of ICIs, adoptive cell therapy, cancer vaccine and CAR-T cell therapy. In addition, we discuss the current challenges of immunotherapies, as well as potential strategies to overcome these limitations, such as combination of immunotherapy and targeted therapy.

## 2. Immunotherapy for Advanced Gastric Cancer

**Figure 1:**
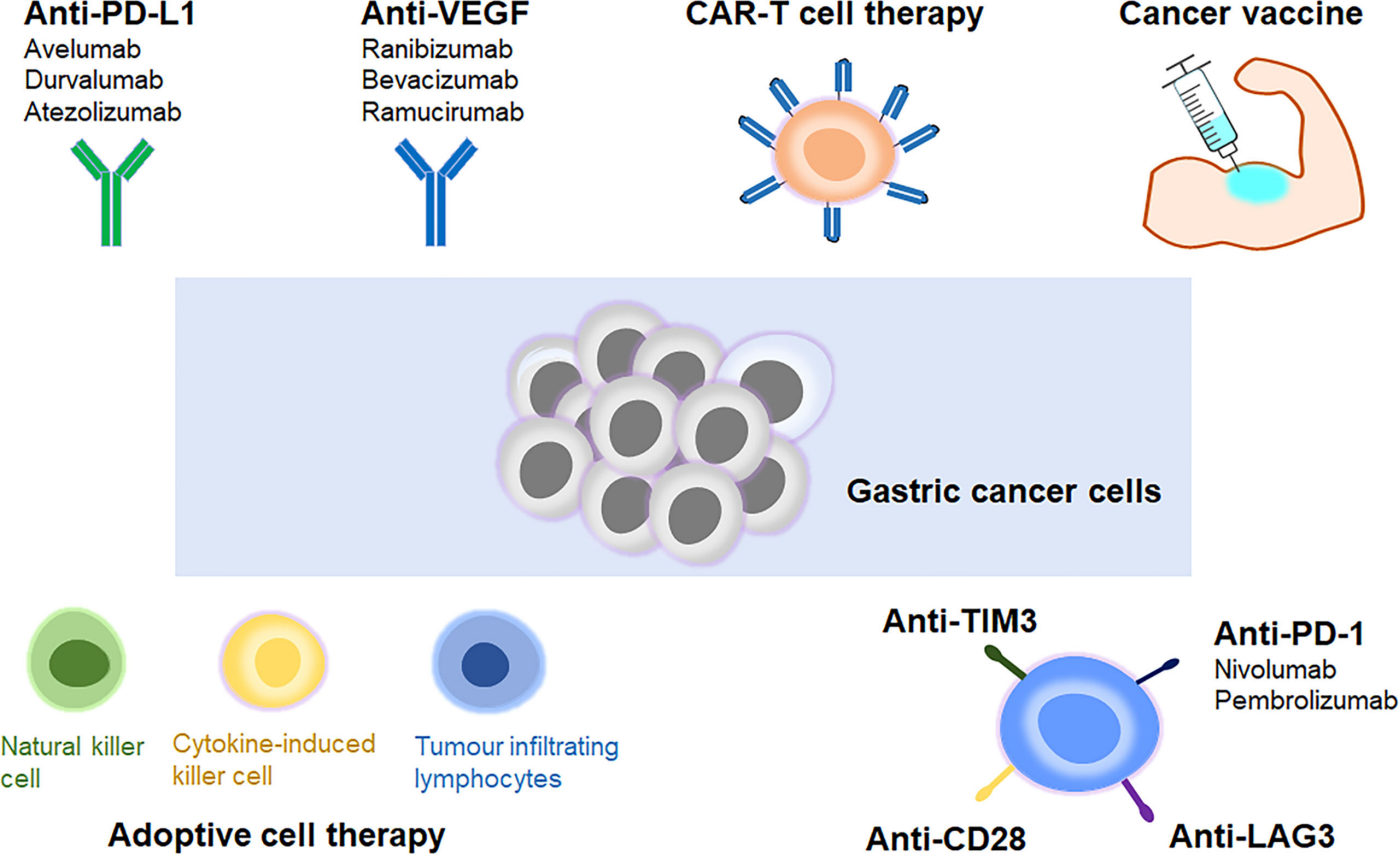
Different types of immunotherapies in advanced gastric cancer. Immune checkpoint inhibitor, adoptive cell therapy, VEGF inhibitor, cancer vaccine and CAR-T cell therapy are the main types of immunotherapies for treatment of advanced gastric cancer.

Over the past few years, a better understanding of the immune mechanism of gastric cancer has greatly facilitated the development of novel immunotherapies. ICIs could effectively interrupt the immune checkpoint interactions, leading to the destruction of tumour cells *via* activation of host’s immune system (16). The ongoing clinical trials of ICIs in advanced gastric cancer have been listed in **Table 1**. Other approaches such as adoptive cell therapy, VEGF inhibitors, cancer vaccines and CART cell therapy have also demonstrated potent anti-tumour activities (11, 17). These achievements in immunotherapy have marked a new era for advanced gastric cancer treatment (**Figure 1**).

**Table 1:** Representative clinical trials of ICIs in advanced gastric cancer.

Agent	Phase	Target	Conditions	NCT number	Other identifier
Ipilimumab	II	CTLA-4	Adenocarcinoma of the Stomach GastroEsophageal Cancer	NCT03647969	AIOSTO-0417
Nivolumab		PD-1			
Ipilimumab	III	CTLA-4	Gastric Cancer Gastroesophageal Junction Cancer	NCT02872116	CheckMate-649
Nivolumab		PD-1			
Atezolizumab	II	PD-L1	Gastric Cancer Gastroesophageal Junction Adenocarcinoma	NCT03421288	DANTE
Durvalumab	II	PD-L1	Advanced solid tumors (including gastric cancer)	NCT04157985	/
Avelumab					
Nivolumab	III	PD-1	Gastroesophageal Junction Cancer	NCT02743494	CheckMate-577
Pembrolizumab	III	PD-1	Gastric Cancer Gastroesophageal Junction Cancer	NCT03221426	KEYNOTE-585
Nivolumab	II	PD-1	Gastric Cancer Cancer of the Stomach Esophagogastric Junction	NCT03662659	CA224-060
Relatlimab		LAG-3			
Pembrolizumab Trastuzumab	III	PD-1	Gastric Neoplasms Gastroesophageal Junction Adenocarcinoma	NCT03615326	KEYNOTE-811
		HER-2			
Nivolumab	II	PD-1	Unresectable advanced or recurrent gastric cancer	NCT02267343	ATTRACTION-2
Pembrolizumab	III	PD-1	Advanced gastric gastroesophageal junction adenocarcinoma	NCT02370498	KEYNOTE-061
Pembrolizumab	III	PD-1	Advanced gastric or gastroesophageal junction adenocarcinoma	NCT02494583	KEYNOTE-062
Pembrolizumab	III	PD-1	Gastric Neoplasms Gastroesophageal Junction Adenocarcinoma	NCT03019588	KEYNOTE-063

### 2.1 Immune Checkpoint Inhibitors

In 2011, ipilimumab became the world-first approved ICIs to treat melanoma (18). Since then, immune therapies have revolutionized the strategies for advanced gastric cancer treatment. There are mainly three types of ICIs, anti-PD1/PD-L1 and anti-CTLA4 antibodies (19). Activated immune cells such as T cells can express PD-1. PD-L1, the ligand of PD-1, binds to PD-1 thus resulted in immune cell apoptosis and immune suppression. PD-L1 is overexpressed in advanced gastric cancer, leading to the evasion of tumour cells from immune response (20). On the other hand, CTLA-4 protein can interact with B7-1/B7-2 with high affinity, leading to CD28 signalling pathway inhibition, which plays a critical role in T cell activation (21). Inhibitors targeting these immune checkpoints have been generated and studied in pre-clinical and clinical trials.

#### 2.1.1 PD-1 Inhibitors

PD-1 inhibitor nivolumab is a monoclonal antibody that have gained the approval of FDA in the year 2014 for advanced gastric tumour treatment (22). The effects of nivolumab against advanced gastric cancer were examined *via* phase III clinical trials conducted over 40 countries in Asian (13). The initial results showed that nivolumab could significantly increase the survival rate of patients compared to the placebo. Nivolumab treatment in gastric tumour patients have demonstrated 12-month overall survival rates of 26.2% in contrast to that of 10.9% survival rates by placebo treatment, suggesting a promising cure for this poor prognosis population. Notably, nivolumab has been approved for clinical application as a novel approach to treat advanced and recurrent gastric cancer (22, 23).

Pembrolizumab is another promising inhibitor targeting PD-1. The efficacy of pembrolizumab has been assessed in the phase II trials among advanced gastric cancer (24). Treatment with pembrolizumab in advanced gastric cancer patients has demonstrated 11-month overall survival rates of 45.8%. In addition to its high anti-tumour activity, pembrolizumab has also shown moderate side effects. These advantages of pembrolizumab have prompted its approval for treatment of advanced gastric tumour in 2017 (25). Clinical trials of pembrolizumab in 592 patients with non-operable advanced gastric cancer have been conducted to determine the efficacy of pembrolizumab in comparison to paclitaxel (26). However, pembrolizumab alone didn’t demonstrate significantly improved survival rates compared to paclitaxel in patients. When pembrolizumab combined with paclitaxel, enhanced anti-tumour effect and better toleration was detected (27). Tislelizumab has also been assessed for its anti-tumour effect against advanced gastric cancer, providing hope for the evolution of PD-1-based immunotherapy in advanced gastric tumour (28).

#### 2.1.2 PD-L1 Inhibitors

PD-L1 is overexpressed on various cancer cells and plays a crucial role in T cell inhibition (29). The well-known PD-L1 inhibitors include avelumab, durvalumab and atezolizumab (30). Avelumab is an anti-PD-L1 mAb which has demonstrated well toleration in the phase III trial among patients with advanced gastric cancer (31). Avelumab among patients from Japan have exhibited high overall response rates and survival rates. In addition, the efficacy of avelumab against advanced gastric cancer is enhanced in combination with other therapeutics. However, a phase I trial in advanced gastric cancer showed that atezolizumab was effective in one case out of 171 patients (10). The response rates in this clinical trial are closely related to PD-L1 expression. The mechanism of how PD-L1 inhibitors contribute to advanced gastric cancer may be that PD-L1 inhibition could activate DC cells, T lymphocytes and natural kill cells, thus leading to the destruction of gastric tumour (32).

#### 2.1.3 CTLA-4 Inhibitors

CTLA-4 plays important roles in human immune system. CTLA-4 is homologous to CD28, but it can interact with B7-1/B7-2 with higher affinity (21). Therefore, CTLA-4 can regulate or even inhibit CD28 signalling. CTLA-4 inhibitors tremelimumab and ipilimumab have been evaluated in clinical trials of advanced gastric cancer (10). Evaluation of ipilimumab was performed in a phase II trial among advanced gastric cancer patients (33). However, this study was terminated because ipilimumab didn’t demonstrate significant improved survival rate compared with first line targeted agents. A clinical study of tremelimumab on 12 patients with non-operable advanced gastric cancer demonstrated moderate response rate, compared with a combined therapy using both tremelimumab and other anti-cancer agents. Of note, combined therapies targeting CTLA-4 and PD-1 have shown enhanced anti-tumour immunity (34). Combination therapy of ipilimumab and nivolumab has been approved to treat advanced gastric cancer. However, the efficacy of CTLA-4 inhibitor as a monotherapy in advanced gastric cancer remains to be further investigated.

### 2.2 Adoptive Cell Therapy

Gastric cancer cells can express specific neoantigens of high immunogenicity, thus leading to the activation of human immune system. In this way, cancer cells can be recognized and destroyed. However, cancer cells can generate suppressive factors including lymphocyte-activation gene 3 (LAG-3), TGF-β, prostaglandin E2 and IL-10 that inhibit immune response, thus escaping detection and clearance by the immune system (35). For patients whose immune systems fail to detect and response to cancer cells, adoptive cell therapy has been proved as effective strategies to treat advanced gastric cancer (36). Adoptive cell therapy utilizes various immune cells including tumour infiltrating lymphocytes (TILs), lymphokine-activated killer cells and cytokine-induced killer (CIK) cells to induce effective immunity to clear cancer cells (2, 37).

CIK cells are derived from peripheral blood lymphocytes in the presence of CD3 monoclonal antibodies, IL-2 and IFN-γ (38). The CIK cell population consists of CD3^+^CD56^-^ T cell and CD3^+^CD56^+^ T cell, with high anti-tumour activity and proliferation activity (39). Moreover, CIK cells could generate cytokines and chemokines for the regulation and elevation of immune response. A preclinical study using CIK cells demonstrated strong anti-tumour activity of CIK (40). In addition, clinical trials of combined therapy using CIK cells and targeted therapy have shown increased effect against advanced gastric cancer (41).

TILs immunotherapy has been widely applied in advanced gastric cancer. In particular, TILs derived from gastric cancer in patients have been exposed to tumour specific antigens thus are extremely advantageous in immunotherapy. Clinical trials of adoptive cell therapy among gastric cancer patients have shown that combined therapies based on tumour-associated lymphocytes could increase the survival rate to 50% in comparation with using traditional therapy alone (42, 43). Furthermore, in recent years, expanded allogenic natural kill cells has also been used as a novel immunotherapy for treatment of advanced gastric cancer (44). Natural kill cells possess high anti-tumour activity and antibody-dependent cytotoxicity. However, the clinical application of natural kill cells in cancer treatment is severely limited by the lack of strategies to obtain a large amounts of functional natural kill cells (45). Further studies will be taken to investigate novel methods to generate sufficient natural kill cells for cancer immunotherapy.

### 2.3 Anti-Angiogenic Therapy

Vascular endothelial growth factor A (VEGFA) play essential roles in the development of gastric cancer *via* its involvement in the formation of new blood vessels, a process termed as angiogenesis (46). VEGFA functions in the modulation of cancer immune response, which could result in escape of tumour cells from the surveillance of the immune system (47). In addition, VEGF can promote the transfer of Treg cells to the sites of tumour. Clinical studies of combined therapies using VEGFA inhibitors and immune check point inhibitors among patients with advanced gastric cancer have shown promising effects, with enhanced anti-tumour effect and reduced toxicity. For instance, bevacizumab and ramucirumab can significantly prevent angiogenesis (48). Clinical studies of combined therapies using bevacizumab and ICIs such as atezolizumab, ramucirumab, durvalumab in advanced gastric cancer patients have shown favourable efficacy (48). These studies suggest that combined therapy using VEGFA inhibitors and ICIs targeting PD-1 or PD-L1 may shed light on the development of effective treatment in advanced gastric cancer.

### 2.4 Cancer Vaccines

Another novel immunotherapy in advanced gastric cancer is the application of cancer vaccines, which can activate immune responses against tumour cells *in vivo* (49, 50). Proteins and peptides are commonly used antigens to trigger immune responses. The most well-studied cancer vaccines are mRNA vaccines, which carries the genetic information of antigen and can translate it into protein rapidly to induce immune response, thus leading to the destruction of cancer cells (51). Studies have revealed that mRNA cancer vaccines showed strong efficacy and moderate side effects compared to traditional chemotherapy or targeted therapy (52). Moreover, combination of cancer vaccines and chemotherapies such as cisplatin and 5-fluorouracil have exhibited significantly enhanced cytotoxicity against tumour cells in preliminary clinical trials (53). A clinical study of HLA-A24 and HLA-A2 peptides examined the peripheral blood mononuclear cells in gastric cancer patients (54). Results showed that 50% of the patients treated with cancer vaccines had increased humoral and cellular response against vaccinated peptides.

### 2.5 CAR-T Cell Therapy

CAR-T cell is specifically designed for the expression of synthetic receptors that can induce T cells to detect specific cancer antigen, leading to the destruction of tumour cells *via* the host’s immunity (55). Biomarkers such as claudin 18.2 (CLDN 18.2), human epidermal growth factor receptor 2 (HER2), mucin 1, natural-killer receptor group 2 (NKG2D), epithelial cell adhesion molecule (EpCAM), mesothelin (MSLN) and carcinoembryonic antigen (CEA) play important roles in the diagnosis and function of gastric cancer (56). Studies have shown that CAR-T therapy can effectively target the above biomarkers for treatment of advanced gastric cancer (**Table 2**) (57).

**Table 2:** Representative clinical studies of CAR-T cell therapies in advanced gastric cancer.

Agent	Phase	Target	Conditions	NCT number
CAR-CLDN18.2 T cells	I	CLDN18.2	CLDN 18.2 positive advanced gastric adenocarcinoma	NCT03159819
CT041	Ib	CLDN18.2	Claudin18.2-positive adenocarcinoma gastroesophageal junction	NCT03874897
CT041	I	CLDN18.2	Gastric Cancer	NCT04404595

HER2 is a surface antigen overexpressed in gastric cancer cells. HER2-postive gastric cancer usually exhibit multi-drug resistance that inhibit the anti-tumour activity of traditional agents. The development of drug resistance severely hampered the treatment of advanced gastric cancer (57). Of note, CAR-T therapy is an effective strategy to overcome the multiple resistance in advanced gastric cancer patients. Notably, studies of HER2 CAR-T therapy demonstrate high affinity for advanced gastric cancer. Clinical studies of CLDN18.2 CAR-T cells in CLDN18.2-positive patients-derived tumour models have demonstrated high anti-tumour activity (58). CA 72-4 is a surface glycoprotein highly expressed in advanced gastric cancer. CAR-T therapy targeting CA 72-4 has shown potent effect in tumour elimination (59). Therefore, CA 72-4 may be a potential target for advanced gastric cancer treatment. Notably, clinical studies in patients showed that CAR-T therapy in combination with other therapeutics displayed enhanced anti-tumour effects (57).

In addition, CAR-T cells targeting B7-H3 and CDH17 have made achievements in cancer treatment. Clinical studies have shown that B7-H3 is overexpressed in the tumour tissues of advanced gastric cancer patients and B7-H3 is strongly correlated to the advancement of gastric cancer. Anti-tumour effect of B7-H3 specific CAR-T cells has been evaluated in patients with advanced gastric cancer and demonstrated significant cytotoxicity against gastric tumour cells (60). CDH17 is a biomarker of gastrointestinal adenocarcinomas and plays key roles in CA^2+^-dependent adhesion switch and Wnt signalling. Recent progress in CAR-T cells targeting CDH17 has shed light on this novel immunotherapy as a potential safe and effective treatment for advanced gastric cancer. Pre-clinical studies using gastrointestinal carcinoma xenografts in mouse models have demonstrated that CDH17CART therapy has strong potency against advanced gastric cancer with no obvious toxicity to normal gastrointestinal epithelial cells (61).

## 3. Challenges and Potential Strategies

Development of immunotherapy in advanced gastric cancer has demonstrated great advantages over traditional therapies. However, there still exists various challenges that have severely limited the clinical application of immunotherapy in advanced gastric cancer, for instance, the side effects and toxicity of ICIs, cancer vaccines and CAR-T therapies.

ICIs can lead to autoimmune toxicities in cancer patients (62, 63). For example, the side effects of nivolumab including fatigue, pruritus and rash. Pembrolizumab treatment in advanced gastric cancer can lead to thyroid-related complications. In addition, ICIs can lead to high risk of transplant loss in patients with organ transplants. Although the side effects of PD-1/PD-L1 and CTLA-4 inhibitors are similar, PD-L1 inhibition can lead to more severe immune adverse events due to the loss of PD-L1 ability to bind to B7 (64). However, these side effects of immune checkpoint inhibition can be effectively prevented by immunosuppressive agents such as corticosteroids without impairing the clinical benefits of immunotherapy in advanced gastric cancer. Furthermore, combination of ICIs and targeted therapy display synergistic effects on advanced gastric cancer.

VEGF has been established as a crucial target for treatment of advanced gastric cancer. However, due to the wide expression of VEGF, side effects of VEGF inhibitors are commonly seen in clinic, including hypothyroidism, coagulation disorders, gastrointestinal perforations, hypertension, proteinuria, neurotoxicity (65).

Although cancer vaccine has shown favourable benefits in phase I and phase II trials against advanced gastric cancer, its clinical efficacy is low because of regulation and suppression from the host immune system. Novel strategies to overcome this limitation involve the development of combined therapies, for example, combination of cancer vaccine and immune modulator to avoid immune suppression, use of conventional chemotherapy in addition to cancer vaccine to enhance anti-tumour activity but reduce cytotoxicity (66).

Despite the amazing efficacy of CAR-T therapy against advanced gastric cancer, this novel treatment also exhibits strong toxicity that could be fatal (57, 67). The most commonly seen side effects of CAR-T therapy are known as cytokine release syndrome and CAR-T therapy-related encephalopathy syndrome that includes fevers, chills, nausea, headache, cardiac toxicity and neurotoxicity. Development of CAR-T cells with shorter lifespan or “on-switch” may effectively overcome the limitations of current CAR-T therapy, reduce the toxicity, and facilitate the wide clinical application of this novel immunotherapy in advanced gastric cancer (68).

## 4. Conclusion

Over the past decades, cancer immunotherapy has emerged as promising therapeutics for various cancers. Development of ICIs has been a breakthrough for advanced gastric cancer and demonstrated anti-tumour effect in patients. However, the toxicity and efficacy of immune checkpoint inhibition have largely limited its broad clinical application. Other immunotherapies including adoptive cell therapy, cancer vaccines and CAR-T cell therapy also showed anti-tumour activity in gastric cancer patients. Clinical trials of immunotherapy in combination with targeted therapy have shown enhanced anti-tumour activity and survival rate compared with using immunotherapy alone. Despite the advantages of immunotherapy in advanced gastric cancer, challenges such as moderate clinical efficacy and immune evasion blocks the broad application of immunotherapy in advanced gastric cancer. New strategies to overcome these challenges will involve combination of CAR-T therapy and ICIs, utilizing of immune modulators to avoid immune suppression. We believe that developing novel immunotherapy may shed lights on the treatment of advanced gastric cancer.

## Author Contributions

DY, KY, and XC conceived the topic, revised and proofread the manuscript. XJ and ZL drafted the paper and prepared the figure and table. All authors approved the submitted version.

## Funding

This study was supported by the Shanghai Science and Technology Funds (21Y11913100).

## Conflict of Interest

The authors declare that the research was conducted in the absence of any commercial or financial relationships that could be construed as a potential conflict of interest.

## Publisher’s Note

All claims expressed in this article are solely those of the authors and do not necessarily represent those of their affiliated organizations, or those of the publisher, the editors and the reviewers. Any product that may be evaluated in this article, or claim that may be made by its manufacturer, is not guaranteed or endorsed by the publisher.
